# Inter-specialty differences in the management of febrile neonates on prostaglandin E1

**DOI:** 10.1038/s41372-025-02512-w

**Published:** 2025-11-24

**Authors:** Sharon Morag, Alexander Lowenthal, Hiba Abuelhija, Uri Pollak

**Affiliations:** 1 Department of Neonatology, Dana Dwek Children’s Hospital, Tel Aviv Sourasky Medical Center, Tel Aviv, Israel; 2 Faculty of Medicine and Health Sciences, Tel Aviv University, Tel Aviv, Israel; 3 Pediatric Cardiology, Dana Dwek Children’s Hospital, Tel Aviv Sourasky Medical Center, Tel Aviv, Israel; 4 Cardiac Intensive Care Unit, Heart and Lung Directorate, Great Ormond Street Hospital for Children NHS Foundation Trust, London, UK; 5 Section of Pediatric Critical Care; Hadassah University Medical Center, Ein Kerem, Jerusalem, Israel; 6 Faculty of Medicine, The Hebrew University of Jerusalem, Jerusalem, Israel

**Keywords:** Risk factors, Outcomes research

## Abstract

**Objective:**

To assess inter-specialty differences in the management of febrile neonates receiving prostaglandin E1 (PGE1) for duct-dependent congenital heart disease.

**Study design:**

A cross-sectional, web-based survey was distributed to 800 neonatologists and pediatric cardiac intensivists in North America, Europe, and the Middle East. Respondents (*n* = 526) were queried regarding their management of a clinical vignette involving a febrile neonate on PGE1, including decisions about sepsis work-up, antibiotic administration, and lumbar puncture. Data were analyzed using chi-square tests and multivariate logistic regression.

**Result:**

Pediatric cardiac intensivists were significantly more likely to initiate full sepsis work-up and antibiotic therapy (65.6% vs. 38.7%, *p* < 0.001). Specialty was the only significant predictor of antibiotic administration (OR = 3.07, 95% CI: 1.76–4.40, *p* < 0.001). No significant differences were found in lumbar puncture practices.

**Conclusion:**

Significant inter-specialty variation exists in the management of febrile neonates on PGE1, highlighting the need for evidence-based guidelines to standardize care and optimize antimicrobial stewardship.

## Introduction

Prostaglandin E1 (PGE1, alprostadil) is a cornerstone therapy in the neonatal management of duct-dependent congenital heart disease (CHD), enabling the maintenance of ductal patency and thereby supporting either systemic or pulmonary circulation until definitive surgical or catheter-based intervention is feasible [1]. In conditions such as pulmonary atresia, critical coarctation of the aorta, or hypoplastic left heart syndrome, the patency of the ductus arteriosus is essential for adequate systemic or pulmonary perfusion [2]. The use of PGE1 in this context has transformed survival outcomes for these neonates. However, while lifesaving, PGE1 is frequently associated with adverse effects, particularly fever, which occurs in up to 40% of treated neonates [3, 4].

Fever in neonates less than 28 days of age remains a clinical red flag, strongly associated with serious bacterial infections including bacteremia, meningitis, and urinary tract infections [5]. Current pediatric and neonatal guidelines advise a full sepsis evaluation, including blood cultures, lumbar puncture (LP), and prompt empiric administration of intravenous antibiotics when fever is present [6–8]. However, in neonates receiving PGE1, the etiology of fever becomes diagnostically challenging. Fever may reflect a benign pharmacologic effect of the medication, an effect previously well-documented [3, 4], or it may represent a true infectious process. This ambiguity poses a complex dilemma: under-treatment risks missing a life-threatening infection, whereas over-treatment may lead to unnecessary invasive procedures, prolonged hospitalization, disruption of cardiac care plans, and contribution to antimicrobial resistance [9–11].

The lack of dedicated evidence or standardized clinical guidelines addressing fever management in neonates treated with PGE1 has led to wide practice variation. Some clinicians may favor a conservative approach, attributing the fever to PGE1 and observing without intervention, while others proceed with a full infectious evaluation and antibiotic therapy to err on the side of caution [4, 10]. In real-world practice, this results in a spectrum of responses ranging from no evaluation at all to full sepsis work-up including LP and broad-spectrum antibiotics. Such variability may be influenced by provider specialty, training, institutional protocols, and regional medical culture.

Differences between neonatologists and pediatric cardiac intensivists may be particularly relevant. For instance, a study by Borenstein-Levin et al. found significant inter-specialty differences in approaches to ventilation, sedation, hemodynamics, and vascular access in the care of the same critically ill neonates [12].

Given the clinical consequences of both under- and over-treatment of febrile neonates, it is important to better understand these practice patterns. This study seeks to evaluate and compare the clinical approaches of neonatologists and pediatric cardiac intensivists regarding the management of fever in neonates receiving PGE1 for duct-dependent CHD. By identifying patterns and predictors of practice variation, this research aims to inform future guidelines, promote more standardized care, and support balanced antimicrobial stewardship in this vulnerable patient population.

## Methods

### Study design and objectives

We conducted a cross-sectional, web-based survey to evaluate inter-specialty differences in the management of febrile neonates receiving PGE1 for ductal patency. The study aimed to compare the diagnostic and therapeutic strategies of neonatologists and pediatric cardiac intensivists regarding sepsis evaluation, antibiotic administration, and LP performance. Additional objectives included exploring whether additional provider characteristics (e.g., specialty, region of practice, years of experience, and gender) predict management decisions in this clinical context.

### Survey development

The survey instrument was developed by a multidisciplinary team of neonatologists and pediatric cardiac intensivists, based on expert consensus and a review of existing literature on neonatal fever management and PGE1 therapy. The survey included both multiple-choice and scaled-response questions organized into the following sections:

- Demographics and professional background
- Clinical practice setting and experience
- Management approaches to a clinical vignette describing a febrile neonate receiving PGE1
- Decision-making regarding sepsis work-up, antibiotic initiation, and lumbar puncture Survey items were pretested for clarity and content validity by a small group of specialists (*n* = 8) not included in the final sample. Modifications were made accordingly.

### Participants and distribution

The target population included board-certified neonatologists and pediatric cardiac intensivists practicing in North America, Europe, and the Middle East. A convenience sample of 800 physicians was invited to participate via professional mailing lists and institutional collaborations. Survey participation was anonymous and voluntary. No personal identifiers were collected to preserve confidentiality.

### Data collection and variables

The survey was administered online using a secure, GDPR-compliant platform. Respondents were asked to indicate:

- Their primary specialty (neonatology or pediatric cardiac intensive care)
- Country and region of practice
- Years of clinical experience
- Gender
- Approach to fever in a neonate receiving PGE1, including:
- Whether a sepsis work-up would be initiated
- Whether antibiotic therapy would be started
- Whether a LP would be performed, and under what clinical criteria Management options were offered as categorical variables (e.g., full work-up with antibiotics, partial work-up without antibiotics, etc.). Responses were coded and entered as a de-identified dataset for analysis.

### Statistical analysis

Descriptive statistics were used to summarize demographic data and response distributions. Categorical variables were presented as counts and percentages. Differences in management strategies between specialties were assessed using Chi-square tests. The threshold for statistical significance was set at *p* < 0.05. Multivariate logistic regression models were constructed to identify independent predictors of: Antibiotic administration, Performance of a full sepsis work-up, Decision to perform a LP. Covariates in the models included specialty, gender, region of practice, and years of clinical experience. Odds ratios (ORs) and 95% confidence intervals (CIs) were reported. All analyses were performed using Python (v3.10) with appropriate statistical libraries (pandas, scipy.stats, and statsmodels).

### Ethical considerations

This study involved no patient-level data and did not require ethical approval under institutional guidelines for anonymous survey-based research. Participation implied consent, and respondents were informed of the anonymous nature of data handling and publication.

## Results

### Survey participation and respondent characteristics

Of the 800 physicians invited to participate, 526 completed the survey, yielding a response rate of 65.8%. The respondents included 282 neonatologists (53.6%) and 244 pediatric cardiac intensivists (46.4%). Participants were geographically distributed across North America (58.6%), Europe (26.6%), and the Middle East (14.8%). Regarding clinical experience, 25.3% reported <5 years, 30.8% had 5–10 years, 28.7% had 11–20 years, and 15.2% had >20 years of experience. Slightly more than half of the respondents were female (53.2%). A summary of the demographic characteristics is presented in Table 1.

**Table 1:** Demographic Characteristics of Survey Respondents (*N* = *526)*.

Characteristic	Category	n	%
**Specialty**	Neonatologist	282	53.6
	Pediatric Cardiac Intensivist	244	46.4
**Geographic Region**	North America	308	58.6
	Europe	140	26.6
	Middle East	78	14.8
**Years of Experience**	<5 years	133	25.3
	5–10 years	162	30.8
	11–20 years	151	28.7
	>20 years	80	15.2
**Gender**	Male	246	46.8
	Female	280	53.2

Table summarizing the demographic characteristics of survey respondents, including specialty, geographic region, years of clinical experience, and gender, showing an approximately even split between neonatologists and pediatric cardiac intensivists, with diverse regional and experience representation.

### Fever management and lumbar puncture practices

Fever management approaches for neonates receiving PGE1 varied significantly by specialty. Pediatric cardiac intensivists were more likely to initiate a full sepsis work-up including antibiotics compared to neonatologists (65.6% vs. 38.7%, χ² = 38.32, *p* < 0.001). Neonatologists more frequently opted for no work-up or partial work-up without antibiotics. Fever management strategies were not significantly influenced by region (χ² = 17.79, *p* = 0.120), gender (χ² = 4.78, *p* = 0.310), or years of experience (χ² = 29.79, *p* = 0.106).

Lumbar puncture decision-making was not significantly different between the specialties (χ² = 5.70, *p* = 0.223). However, pediatric cardiac intensivists were more likely to perform LP based on laboratory findings or routine protocols, while neonatologists more often cited systemic signs or clinical deterioration. Differences in LP triggers were not statistically significant across region (χ² = 20.79, *p* = 0.139), gender (χ² = 6.83, *p* = 0.234), or experience (χ² = 23.40, *p* = 0.183). A detailed comparison of fever management and LP practices by specialty is presented in Table 2.

**Table 2:** Fever Management Practices by Medical Specialty.

1. Management Strategy	Neonatologists (*n* = 282)	Cardiac Intensivists (*n* = 244)	*p*-value
**Sepsis Evaluation Approach**			
Full sepsis work-up + empiric antibiotics	109 (38.7%)	160 (65.6%)	<0.001*
Full sepsis work-up, no antibiotics	45 (16.0%)	35 (14.3%)	0.660
Partial work-up, no antibiotics	79 (28.0%)	76 (31.0%)	0.002*
No work-up	65 (23.0%)	89 (36.5%)	<0.001*
**Lumbar Puncture Indications**			
Clinical deterioration	46 (16.3%)	37 (15.2%)	0.723
Abnormal laboratory results	49 (17.4%)	56 (23.0%)	0.135
Systemic signs of infection	53 (18.8%)	49 (20.1%)	0.709
Routine practice	56 (19.9%)	56 (23.0%)	0.412
Other indications	78 (27.7%)	46 (18.9%)	0.010*

Table comparing fever management strategies between neonatologists and pediatric cardiac intensivists, highlighting that cardiac intensivists are significantly more likely to initiate a full sepsis work-up with antibiotics, while neonatologists more often choose conservative approaches; also includes triggers for lumbar puncture by specialty.

^*^Statistically significant (*p* < 0.05).

### Predictors of antibiotic use and full work-up

Antibiotic Administration. Multivariate logistic regression identified specialty as a significant predictor of antibiotic administration. Pediatric cardiac intensivists had 3.07 times higher odds of initiating antibiotics for a febrile neonate receiving PGE1 compared to neonatologists (OR = 3.07; 95% CI, 1.76–4.40; *p* < 0.001). Gender, region, and clinical experience were not statistically significant predictors (Table 3).

**Table 3:** Multivariable Predictors of Empiric Antibiotic Administration.

Predictor Variable	Odds Ratio	95% Confidence Interval	*p*-value
**Specialty**			
Cardiac Intensivist vs. Neonatologist	3.07	1.76 - 4.40	<0.001*
**Clinician Gender**			
Female vs. Male	1.03	0.73 - 1.46	0.878
**Geographic Region**			
North America vs. Other regions	1.22	0.86 - 1.76	0.270
**Clinical Experience**			
>10 years vs. <5 years	0.94	0.60 - 1.49	0.806

Table presenting the results of multivariable logistic regression analysis for predictors of empiric antibiotic administration, demonstrating that pediatric cardiac intensivist specialty is associated with more than three times higher odds of prescribing antibiotics compared to neonatologists, with other factors not statistically significant.

^*^Statistically significant (*p* < 0.05).

Full Sepsis Work-up. Similar results were found for the likelihood of performing a full sepsis work-up, with pediatric cardiac intensivists and clinicians from North America demonstrating significantly increased odds (OR = 2.92; 95% CI, 1.74–4.21; *p* < 0.001). Other variables were not significant.

Lumbar Puncture. No variables, including specialty, gender, region, or experience, were independently associated with LP performance in the multivariate analysis.

## Discussion

This cross-sectional survey reveals significant inter-specialty differences in the management of febrile neonates receiving PGE1 therapy. Our findings demonstrate that pediatric cardiac intensivists are substantially more likely than neonatologists to initiate full sepsis workups with empiric antibiotic therapy, with more than three-fold increased odds of antibiotic administration. These results highlight a critical gap in standardized care for this vulnerable population and underscore the need for evidence-based guidelines to optimize clinical decision-making.

### Clinical implications and patient safety considerations

The substantial practice variation identified in our study raises important questions about optimal care for febrile neonates receiving PGE1. While neonates with CHD have higher risk of sepsis [13], both over-treatment and under-treatment may carry significant risks. Aggressive sepsis evaluation and empiric antibiotic therapy may lead to unnecessary invasive procedures, prolonged hospitalization, increased healthcare costs, and contribution to antimicrobial resistance [14, 15]. LP in neonates with complex congenital heart disease may pose additional procedural risks, particularly in those with ductal-dependent circulation where hemodynamic instability could be precipitated by positioning or procedural stress [16].

Conversely, conservative management risks missing life-threatening bacterial infections. Early-onset sepsis remains a leading cause of neonatal morbidity and mortality, with case fatality rates of 10–15% for bacteremia and up to 25% for meningitis [5, 17]. The immunocompromised state of critically ill neonates with congenital heart disease may further increase susceptibility to serious bacterial infections [18].

The absence of statistically significant differences in LP practices between specialties, despite differences in overall sepsis work-up approaches, suggests that both groups recognize the procedural risks and complexity of LP in this population. However, the variation in clinical triggers for LP (laboratory findings versus clinical deterioration) indicates ongoing uncertainty about optimal indications.

### Factors influencing practice patterns

Our analysis revealed that demographic factors such as clinician gender, years of experience, and geographic region were not significant predictors of management decisions. This suggests that specialty-specific training and culture, rather than individual clinician characteristics, are the primary drivers of practice variation. The lack of regional differences is somewhat surprising given documented variations in antibiotic prescribing patterns and sepsis management protocols across different healthcare systems [19, 20].

The absence of experience-related differences indicates that practice patterns are established early in specialty training and remain consistent throughout career progression. This finding supports the importance of evidence-based education and standardized protocols during residency and fellowship training programs.

### Toward evidence-based guidelines

The substantial practice variation documented in our study underscores the urgent need for evidence-based guidelines specific to fever management in neonates receiving PGE1. Current pediatric sepsis guidelines do not address the unique clinical context of drug-induced fever in critically ill neonates with congenital heart disease [21, 22].

Several strategies could help optimize care for this population. First, development of clinical prediction rules incorporating patient-specific factors (hemodynamic stability, biomarkers, duration of PGE1 therapy, etc.) could guide risk stratification and management decisions [23]. Second, implementation of standardized protocols that balance infection risk with the potential harms of over-treatment could reduce practice variation while maintaining safety [24]. Third, prospective studies examining outcomes associated with different management approaches could provide evidence to inform future guidelines.

Recent studies have begun to address this knowledge gap in managing fever in neonates receiving PGE1. Baranwal et al. reported in a prospective study that presepsin may help distinguish PGE1-induced systemic inflammation from true bacterial sepsis, offering a biomarker that could curb unnecessary antibiotic exposure in this population [25]. In parallel, C-reactive protein (CRP) and procalcitonin (PCT) have shown utility in early-onset neonatal sepsis and, when interpreted in the context of PGE1 therapy, may help differentiate infectious from non-infectious fever [26, 27]. Notably, the delayed rise of CRP limits its value for early rule-in decisions within the first 12–24 h, whereas serial CRP trends can support de-escalation once available; by contrast, the earlier kinetics of PCT and presepsin may facilitate initial risk-stratification and thereby reduce unnecessary lumbar punctures and empiric antibiotics when embedded in standardized pathways [25–27]. Prospective validation is needed to define clinically actionable cut-offs and to clarify how biomarker-guided algorithms should interface with hemodynamic status and local epidemiology.

### Future research directions

Our findings highlight several areas for future investigation. Prospective cohort studies examining clinical outcomes associated with different management strategies would provide critical evidence for guideline development. Additionally, research into biomarkers that can reliably distinguish PGE1-induced fever from infectious fever could revolutionize clinical decision-making in this population.

Cost-effectiveness analyses comparing aggressive versus conservative management approaches could inform resource allocation and policy decisions. Finally, implementation studies evaluating the effectiveness of standardized protocols in reducing practice variation while maintaining patient safety would be valuable for healthcare systems seeking to optimize care quality.

### Limitations

Several limitations should be considered when interpreting our findings. First, the cross-sectional survey design captures reported practice patterns rather than actual clinical outcomes, limiting our ability to determine which approach leads to better patient outcomes. The clinical vignette methodology, while standardized, may not fully capture the complexity of real-world decision-making where multiple clinical factors influence management choices. Second, selection bias may have influenced our results, as physicians who chose to participate in the survey may differ systematically from non-respondents in their practice patterns or interest in evidence-based medicine. The 65.8% response rate, while reasonable for physician surveys, leaves open the possibility that non-respondents have different practice patterns. Third, our convenience sampling approach may not fully represent the broader population of neonatologists and pediatric cardiac intensivists. The geographic distribution of respondents, while spanning three continents, may not reflect global practice patterns, particularly in resource-limited settings where diagnostic capabilities and antibiotic availability may influence management decisions. Fourth, the survey instrument, despite pretesting and expert review, may not have captured all relevant clinical factors that influence decision-making in practice. Social desirability bias may have led some respondents to report practices that align with perceived best practices rather than their actual clinical behavior. Finally, our study did not collect data on institutional protocols or local guidelines that may influence individual clinician decision-making. Practice variation may be partially explained by differences in institutional culture and protocols rather than individual clinician preferences alone.

## Conclusions

This multinational survey demonstrates significant inter-specialty differences in the management of febrile neonates receiving PGE1 therapy, with pediatric cardiac intensivists substantially more likely to pursue aggressive sepsis evaluation and empiric antibiotic therapy compared to neonatologists. These findings highlight the absence of evidence-based guidelines for this clinical scenario and underscore the need for standardized approaches that balance infection risk with the potential harms of over-treatment.

The substantial practice variation identified in our study represents a serious gap in pediatric healthcare that requires appropriate attention. Development of evidence-based clinical guidelines, informed by prospective outcome studies and validated risk stratification tools, is essential to optimize care for this vulnerable population. Such guidelines should incorporate specialty-specific perspectives while prioritizing patient safety and antimicrobial stewardship principles.

Future research should focus on prospective studies examining clinical outcomes associated with different management strategies, development of biomarkers to distinguish infectious from drug-induced fever, and implementation of standardized protocols that can reduce practice variation while maintaining high-quality care. Only through such collaborative efforts can we ensure that neonates with life-threatening congenital heart disease receive optimal, evidence-based care during their most vulnerable period.

## Data Availability

The de-identified dataset supporting the conclusions of this article is available from the corresponding author (Dr. Uri Pollak, uripol@hadassah.org.il) upon reasonable request for legitimate research purposes. Requests must include a detailed research proposal, institutional affiliation verification, and appropriate ethical approval where required. Data will be shared in standard formats with documentation, subject to conditions ensuring participant confidentiality, data security, and appropriate attribution of the original study.
